# Evolution and Determinants of Lung Function until Late Infancy among Infants Born Preterm

**DOI:** 10.1038/s41598-019-57359-x

**Published:** 2020-01-16

**Authors:** Shen-Hao Lai, Ming-Chou Chiang, Shih-Ming Chu, Jen-Fu Hsu, Tsung-Chieh Yao, Ming-Han Tsai, Man-Chin Hua, Chih-Yung Chiu, Kuo-Wei Yeh, Jing-Long Huang, Sui-Ling Liao

**Affiliations:** 1 Department of Pediatrics, Chang Gung Memorial Hospital Linkou Branch, Taoyuan, Taiwan; 2 Department of Pediatrics, Chang Gung University, Taoyuan, Taiwan; 3 Department of Pediatrics, Chang Gung Memorial Hospital Keelung Branch, Keelung, Taiwan; 4 Prediction of Allergies in Taiwanese Children (PATCH) cohort study, Keelung, Taiwan

**Keywords:** Respiratory tract diseases, Outcomes research

## Abstract

To investigate the evolution of lung function in preterm infants with and without bronchopulmonary dysplasia (BPD) and to determine the perinatal characteristics associated with indexes of lung function in later infancy. Longitudinal lung function assessments were performed at approximately 6, 12, 18, and 24 months of corrected age in preterm infants. Perinatal characteristics were further analyzed to ascertain the determinants of lung function indexes. Although all preterm infants (n = 121; 61 without BPD and 60 with BPD) exhibited decreased lung function in early infancy (6 months of age), after body length was adjusted for, only infants with BPD exhibited poor performance. Furthermore, the lung function of infants with mild to moderate BPD caught up gradually, but the generally poor lung function performance of infants with severe BPD, especially in forced expiratory flow, persisted until later age (24 months). Regarding perinatal characteristics, the z-score of body length at the time of examination and total number of days on positive-pressure ventilation are the major determinants of lung function in later infancy.

## Introduction

Bronchopulmonary dysplasia (BPD) is a chronic lung disease that develops in preterm infants, namely less than 37 weeks’ gestational age, who were exposed to mechanical ventilation and oxygen supplementation. Dysplasia of microvessels, airways, and alveoli have been observed in lungs of such premature infants^1^. The “new” form of BPD, deemed a developmental disorder, differs pathologically and clinically from the “old” form^2^. Before the period of antenatal steroid and intratracheal surfactant, the “old” BPD occurred in preterm infants (usually late preterm) who suffered from surfactant deficiency. These infants required high-ratio oxygen and ventilatory support, which trigger a heterogenous lung injury and remodeling. The sequels of lung damage marks with regions of atelectasis and hyperinflation, hyperplasia of airway smooth muscle, lung fibrosis, and pulmonary vascular hypertensive changes^3^. After the period of prenatal steroid and postnatal surfactant intervention, the mortality of BPD significantly decreased, and shifted the demographics of BPD to more extreme preterm infants. “New” BPD then occurred and being characterized by arrested alveolar-capillary development, namely fewer and larger alveoli develop, reducing the overall surface available for gas exchange^4^. The airways are relatively less affected, and inflammation is usually less prominent than in the old form of BPD^1^.

Although previous studies have showed that infants with BPD have significant decline in lung function till third decade of life^5^, it’s uncertain whether this defect is a consequence of prematurity and/or BPD. Long-term studies on the lung function of survivors of old BPD have reported that abnormalities in forced expiratory flow persisted over time^6–8^. However, the results from these studies may differ from children with new BPD, which usually develops in more preterm infants, who might have received markedly different respiratory care. Recent studies that have enrolled infants with new BPD have found persistent impairment of lung function^9–13^, but the cohorts in most of these studies were small; in addition, these studies have not investigated the determinants of various indexes of lung function.

Nowadays, in the age of “new” BPD (with the intervention of postnatal surfactant treatment), through this longitudinal cohort study, we first investigated the differences in the lung function of infants with BPD of various levels of severity and assessed the evolution of lung function by BPD severity. Second, we analyzed the perinatal determinants of various lung function indexes at different time points in later infancy.

## Results

During the study period, a cohort of 121 infants was enrolled. Table 1 presents the demographic characteristics at birth and postnatal care of infants with BPD of various levels of severity. Infants with relatively higher BPD severity had relatively lower gestational age and birth weight; the proportion of surfactant delivery was higher and duration (days) of positive-pressure ventilation was longer (p < 0.001).

**Table 1:** Demographic characteristics of infants stratified by severity of bronchopulmonary dysplasia (BPD).

	Total (n = 121)	No BPD (n = 61)	Mild-moderate BPD (n = 34)	Severe BPD (n = 26)
GA, wk	30 ± 3 (23–34)	32 ± 1 (28–34)	28 ± 2 (23–33)*	26 ± 1 (23–28)*^#^
Birth weight, g	1422 ± 513 (476–2504)	1844 ± 323 (1064–2504)	1183 ± 302 (630–2094)*	851 ± 218 (476–1290) *^#^
Boys	63 (52.1)	35 (60.3)	17 (50)	9 (34.6)
PROM	41 (33.9)	19 (32.7)	11 (32.3)	11 (42.3)
Prenatal steroid	71 (58.6)	25 (43.1)	28 (72.3)	22 (84.6)^#^
Surfactant	32 (26.4)	0 (0)	14 (41.1)*	15 (57.7)*
Duration of positive-pressure ventilation, d	39 ± 55 (0–420)	6 ± 1 (0–31)	43 ± 21 (18–90)*	114 ± 78 (55–420) *^#^

*p < 0.001 compared with the no BPD group; ^#^ p < 0.001 compared with the mild to moderate BPD group.

PROM, premature rupture of membrane.

Values were presented as means and standard deviations (ranges) or numbers (proportions, %).

Table 2 presents the data of infants receiving various lung function examinations at different time points, including the absolute values and z-scores of somatic characteristics (i.e., body weight and body length). Infants without BPD persistently demonstrated relatively normal somatic growth. Table 2 showed that the growth of infants with mild to moderate BPD gradually caught up (especially in body weight), but significantly poor somatic growth in terms of body length persisted in infants with severe BPD until 24 months of corrected age (p < 0.05).

**Table 2:** Demographic characteristics of infants at age 6, 12, 18, and 24 months stratified by severity of bronchopulmonary dysplasia (BPD).

	No BPD	Mild-moderate BPD	Severe BPD
*6 mo*			
No. of children (n)	39	38	15
Body weight, kg	7.54 ± 0.91	7.63 ± 0.82	7.27 ± 1.57
Body weight, z-score	−0.27 ± 1.05	−0.13 ± 0.80	−0.94 ± 1.30
Body length, cm	66.9 ± 3.3	67.1 ± 2.7	66.2 ± 3.4
Body length, z-score	−0.0.07 ± 1.18	−0.09 ± 0.99	−0.97 ± 1.00*^#^
Tidal breathing analysis (n)	39	38	15
Respiratory mechanics (n)	37	31	14
Tidal forced expiration (n)	32	32	14
Raised-volume forced expiration (n)	10	15	10
*12 mo*			
No. of children	37	22	16
Body weight, kg	9.49 ± 0.98	8.90 ± 1.33	8.85 ± 1.32
Body weight, z-score	−0.05 ± 0.90	−0.53 ± 1.16	−0.77 ± 1.06*
Body length, cm	76.6 ± 2.8	74.6 ± 3.5**	74.8 ± 3.5*
Body length, z-score	0.26 ± 1.09	−0.36 ± 1.05*	−0.51 ± 0.86*
Tidal breathing analysis (n)	37	22	16
Respiratory mechanics (n)	30	21	14
Tidal forced expiration (n)	33	22	15
Raised-volume forced expiration (n)	10	10	10
*18 mo*			
No. of children	30	14	14
Body weight, kg	10.84 ± 1.13	9.83 ± 1.63*	10.41 ± 1.37
Body weight, z-score	−0.03 ± 0.82	−0.72 ± 1.09*	−0.63 ± 1.02*
Body length, cm	82.0 ± 2.8	79.3 ± 4.7*	81.1 ± 3.3
Body length, z-score	−0.19 ± 0.86	−0.87 ± 1.11*	−0.86 ± 0.91*
Tidal breathing analysis (n)	30	14	14
Respiratory mechanics (n)	24	14	14
Tidal forced expiration (n)	29	14	12
Raised-volume forced expiration (n)	12	10	12
*24 mo*			
No. of children	16	11	10
Body weight, kg	12.35 ± 1.63	11.95 ± 1.67	11.18 ± 2.31
Body weight, z-score	−0.01 ± 0.93	−0.36 ± 1.02	−0.85 ± 1.83
Body length, cm	88.4 ± 3.6	83.9 ± 4.3*	85.3 ± 4.7
Body length, z-score	0.04 ± 1.04	−1.50 ± 1.22**	−0.71 ± 1.48*
Tidal breathing analysis (n)	16	11	10
Respiratory mechanics (n)	14	10	10
Tidal forced expiration (n)	14	10	10
Raised-volume forced expiration (n)	10	8	8

*p < 0.05 compared with the no BPD group; **p < 0.01 compared with the no BPD group

^#^p < 0.05 compared with the mild to moderate BPD group.

To compare the lung function indexes of preterm and term healthy infants, data of the control population from our previous studies were included in the analysis^13,14^. In infants with no BPD, although poor performance was initially observed in some indexes of lung function (Vt, Crs, V’max_FRC_, FEV_0.5_, and FEF_25–75_), these indexes had eventually normalized later in life (Table 3). By contrast, infants with BPD exhibited poor performance in all indexes in early infancy, namely 6 months of age (p < 0.001). As these infants grew older, their tidal breathing and respiratory mechanics performance gradually caught up, but forced expiratory flow (tidal and raised-volume) performance was persistently poor even until 24 months of age (p < 0.01 to 0.001) (Table 3).

**Table 3:** Absolute values of lung function indexes in healthy term controls, premature infants without bronchopulmonary dysplasia (BPD), and premature infants with BPD at different corrected ages.

		Control	Premature without BPD	Premature with BPD
Vt (ml)	*6 mo*	64.74 ± 8.35 (n = 86)	60.47 ± 11.62* (n = 39)	58.41 ± 9.33*** (n = 53)
	*12 mo*	88.23 ± 13.67 (n = 44)	88.46 ± 11.5 (n = 37)	74.09 ± 11.17*** (n = 38)
	*18 mo*	107.4 ± 14.6 (n = 20)	102.7 ± 13.9 (n = 30)	95.0 ± 14.8 (n = 28)
	*24 mo*	114.5 ± 8.3 (n = 10)	117 ± 14 (n = 16)	97.9 ± 22 (=21)
Rrs (kPa*s/L)	*6 mo*	4.31 ± 1.31 (n = 86)	4.65 ± 1.48 (n = 37)	5.43 ± 1.54*** (n = 45)
	*12 mo*	3.24 ± 0.93 (n = 42)	3.24 ± 0.82 (n = 30)	4.06 ± 1.73** (n = 35)
	*18 mo*	2.53 ± 0.45 (n = 19)	3.22 ± 1.05* (n = 24)	4.34 ± 2.56** (n = 26)
	*24 mo*	2.1 ± 0.9 (n = 10)	2.217 ± 0.39 (n = 14)	3.182 ± 1.29* (n = 20)
Crs (ml/kPa)	*6 mo*	102.7 ± 29.9 (n = 86)	84.5 ± 18.4*** (n = 37)	75.2 ± 23.3*** (n = 45)
	*12 mo*	140.9 ± 34.6 (n = 42)	122.4 ± 42.2* (n = 30)	101.1 ± 19.62*** (n = 35)
	*18 mo*	155.2 ± 25.4 (n = 19)	150.3 ± 25.5 (n = 24)	136.7 ± 30.2* (n = 26)
	*24 mo*	180 ± 33 (n = 10)	188 ± 41 (n = 14)	129.4 ± 50** (n = 20)
V’max_FRC_ (ml/s)	*6 mo*	202.9 ± 98.3 (n = 97)	112.8 ± 54.2*** (n = 32)	101.7 ± 52.1*** (n = 46)
	*12 mo*	282.5 ± 117.2 (n = 68)	227.4 ± 88.8* (n = 33)	150.4 ± 65.6*** (n = 37)
	*18 mo*	354.7 ± 134.1 (n = 33)	291.7 ± 110.8* (n = 29)	203.5 ± 102.9*** (n = 26)
	*24 mo*	360.1 ± 135.2 (n = 27)	399.6 ± 136.7 (n = 14)	220.2 ± 138.1** (n = 20)
FEV_0.5_ (ml)	*6 mo*	235 ± 19 (n = 33)	209.5 ± 29** (n = 10)	198.7 ± 31.2*** (n = 25)
	*12 mo*	295.8 ± 17.2 (n = 25)	302.0 ± 25.1 (n = 8)	242.2 ± 50.1*** (n = 20)
	*18 mo*	327.6 ± 19.9 (n = 25)	322.7 ± 33.0 (n = 12)	294.3 ± 57.7** (n = 22)
	*24 mo*	368 ± 26.5 (n = 14)	363.3 ± 46.1 (n = 10)	282.7 ± 56.4*** (n = 16)
**FVC (ml)**	*6 mo*	308.8 ± 39.2 (n = 33)	285.2 ± 49.2 (n = 10)	273.8 ± 55.7** (n = 25)
	*12 mo*	436.4 ± 36.0 (n = 25)	469 ± 42 (n = 8)	355 ± 63.8*** (n = 20)
	*18 mo*	503.1 ± 41.8 (n = 25)	501.7 ± 56.3 (n = 12)	454.5 ± 88.0* (n = 22)
	*24 mo*	587.9 ± 55.7 (n = 14)	582.8 ± 99.4 (n = 10)	440.2 ± 107.7*** (n = 16)
FEF_25–75_ (ml/s)	*6 mo*	427.6 ± 29.4 (n = 33)	364.2 ± 53.2*** (n = 10)	355 ± 60.5*** (n = 25)
	*12 mo*	523.4 ± 27.1 (n = 25)	515.5 ± 51.5 (n = 8)	387.7 ± 66.6*** (n = 20)
	*18 mo*	573.4 ± 31.3 (n = 25)	554.1 ± 59.5 (n = 12)	503.8 ± 103.8** (n = 22)
	*24 mo*	637.1 ± 41.8 (n = 14)	626.4 ± 77.1 (n = 10)	478.3 ± 108.6*** (n = 16)

*p < 0.05 compared with the control group; **p < 0.01 compared with the control group; ***p < 0.001 compared with the control group.

Vt, tidal volume; Rrs, resistance of respiratory system, Crs, compliance of respiratory system; V’max_FRC_, maximal flow at functional residual capacity; FEV_0.5_, forced expiratory volume at 0.5 s; FVC, forced vital capacity; FEF_25–75_, forced midexpiratory flow.

**Figure 1:**
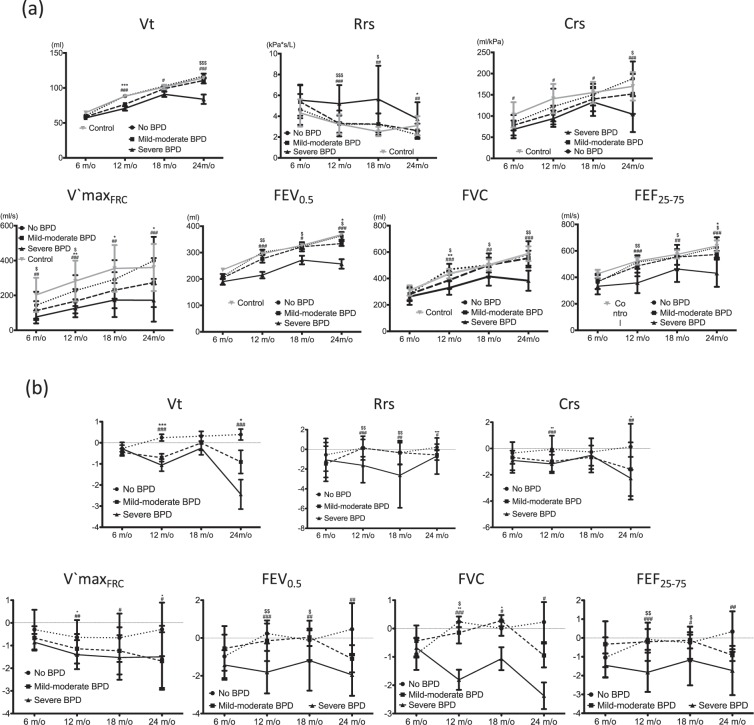
Evolution of various lung-function indexes: (**a**) absolute values and (**b**) z-scores.

To determine the evolution of lung function in infants with BPD of various severity levels, we classified preterm infants into the following groups: no BPD, mild to moderate BPD, and severe BPD. Nearly all indexes of infant lung function testing showed rapid improvement at follow-up. At 24 months of age, except for infants with severe BPD—who exhibited unchanged (or even poorer) performance—the absolute values of all lung function indexes exhibited a gradual increasing trend (Fig. 1). Compared with infants with no BPD and mild to moderate BPD, infants with severe BPD had significantly lower Vt and Crs, higher Rrs, and lower indexes of tidal and raised-volume forced expiration at every measured time point (until 24 months of age); furthermore, respiratory function was negatively correlated with BPD severity.

Table 4 and Fig. 1 present the scores and trends of the respiratory function of infants with BPD of various severity levels obtained after z-score transformation of the aforementioned indexes by using the local healthy refs. ^14,15^. Children with severe BPD in their infancy persistently exhibited poor performance; their z-scores of respiratory function (V’max_FRC_, FEV_0.5_, FVC, and FEF_25–75_) deteriorated with age (18 and 24 months of age). Although children with mild to moderate BPD had relatively lower lung function in early life, their performance (except for V’max_FRC_) gradually improved later in life. Moreover, children without BPD in infancy initially exhibited lower values in lung function (especially in V’max_FRC_, FEV_0.5_, FVC, and FEF_25–75_), but their status improved by 12–18 months of age.

**Table 4:** *Z*-scores of lung function at different corrected ages stratified by the severity of bronchopulmonary dysplasia (BPD).

*z-score*		No BPD	Mild-moderate BPD	Severe BPD
**Vt**	*6 m*	−0.32 ± 0.88	−0.43 ± 0.82	−0.21 ± 0.94
	*12 m*	0.24 ± 0.96	−0.71 ± 0.87***	−1.06 ± 1.11***
	*18 m*	0.31 ± 1.24	−0.01 ± 1.30	−0.25 ± 1.18
	*24 m*	0.39 ± 0.95	−051 ± 1.49*	−1.18 ± 1.69**^#^
**Rrs**	*6 m*	−0.57 ± 1.54	−1.33 ± 1.56	−1.35 ± 1.57
	*12 m*	0.13 ± 0.87	0.12 ± 1.21	−1.62 ± 1.76***^##^
	*18 m*	−0.34 ± 1.16	−0.34 ± 1.21	−2.63 ± 3.28**^##^
	*24 m*	0.24 ± 0.56	−0.37 ± 0.65*	−0.73 ± 1.56*
**Crs**	*6 m*	−0.42 ± 0.81	−0.67 ± 1.03	−0.79 ± 0.76
	*12 m*	−0.06 ± 1.04	−0.99 ± 0.76**	−1.17 ± 0.70***
	*18 m*	−0.27 ± 1.04	−0.67 ± 0.89	−0.52 ± 1.30
	*24 m*	0.35 ± 1.81	−1.44 ± 1.82*	−1.88 ± 1.45**
**V’max**_**FRC**_	*6 m*	−0.43 ± 0.86	−0.74 ± 0.48	−0.84 ± 0.37*
	*12 m*	−0.64 ± 0.76	−1.14 ± 0.65*	−1.40 ± 0.64**
	*18 m*	−0.66 ± 1.07	−1.24 ± 1.04	−1.53 ± 0.98*
	*24 m*	−0.25 ± 1.24	−1.36 ± 1.22*	−1.65 ± 1.17*
**FEV**_**0.5**_	*6 m*	−0.94 ± 0.83	−0.75 ± 0.80	−0.99 ± 0.99
	*12 m*	0.24 ± 0.53	−0.15 ± 1.07	−1.81 ± 1.12***^##^
	*18 m*	−0.13 ± 1.04	0.03 ± 0.46	−1.19 ± 1.59**^#^
	*24 m*	0.13 ± 1.43	−0.97 ± 0.64	−1.89 ± 0.88**
**FVC**	*6 m*	−0.70 ± 0.87	−0.31 ± 0.99	−0.39 ± 1.12
	*12 m*	0.74 ± 0.39	−0.39 ± 0.89**	−1.24 ± 0.86***^#^
	*18 m*	0.01 ± 0.90	0.27 ± 0.59	−1.06 ± 1.33*^#^
	*24 m*	0.20 ± 1.90	−0.60 ± 0.92	−2.07 ± 0.94**^#^
**FEF**_**25–75**_	*6 m*	−1.00 ± 0.79	−0.68 ± 0.89	−1.05 ± 0.78
	*12 m*	−0.08 ± 0.55	−0.20 ± 1.01	−1.82 ± 1.04***^##^
	*18 m*	−0.79 ± 0.85	−0.13 ± 0.43	−1.17 ± 1.34*^#^
	*24 m*	0.01 ± 1.16	−0.85 ± 0.28	−1.79 ± 1.01**

*p < 0.05 compared with the no BPD group; **p < 0.01 compared with the no BPD group; ***p < 0.001 compared with the no BPD group

#p < 0.05 compared with the mild to moderate BPD group; ^##^p < 0.01 compared with the mild to moderate BPD group.

Vt, tidal volume; Rrs, resistance of respiratory system, Crs, compliance of respiratory system; V’max_FRC_, maximal flow at functional residual capacity; FEV_0.5_, forced expiratory volume at 0.5 s; FVC, forced vital capacity; FEF_25–75_, forced midexpiratory flow.

The multivariable GEE analysis discovered that several perinatal and postnatal variables were associated with the performance of lung function (Table 5). In general, total number of days on positive-pressure ventilation had significantly inverse correlation with the z-score of tidal volume (p = 0.008), respiratory mechanics (p = 0.037), and raised forced expiration (p < 0.001). Regarding the somatic variables (body length, body weight, and body mass index), only the z-score of body length at testing time was closely associated with the parameters of raised-volume forced expiration (p around 0.027 to < 0.001). Interestingly, except for tidal volume (p = 0.014), BPD severity had little contribution for other parameters of lung function. Tidal volume (p = 0.006) resistance of respiratory system (p = 0.037) were positively related with gestational age at birth. Regarding the performance of tidal forced expiration, there were no association between these variables. Furthermore, there is no significant association between sex and all parameters of lung function.

**Table 5:** Multivariable GEE analysis of various indexes of lung function and clinical determinants.

*Z scores of indexes*	Clinical determinants	β (SE)	*P*
**Vt**	***BPD***	***−0.116 (0.476)***	***0.014***
	***GA***	***0.326 (0.117)***	***0.006***
	***Vent days***	***−0.012 (0.004)***	***0.008***
	Z-BL	−0.206 (0.228)	0.367
	PROM	0.515 (0.499)	0.302
**Rrs**	BPD	−0.146 (0.243)	0.550
	***GA***	***0.191 (0.092)***	***0.037***
	Vent days	0.003 (0.002)	0.196
	Z-BL	−0.083 (0.107)	0.439
	PROM	−0.476 (0.366)	0.194
**Crs**	BPD	0.212 (0.381)	0.577
	GA	0.022 (0.064)	0.736
	***Vent days***	***−0.007 (0.003)***	***0.048***
	Z-BL	−0.164 (0.200)	0.411
	PROM	−0.078 (0.483)	0.871
**V’max**_**FRC**_	BPD	−0.051 (0.189)	0.787
	GA	0.074 (0.110)	0.503
	Vent days	−0.002 (0.0016)	0.249
	Z-BL	−0.135 (0.278)	0.628
	PROM	−0.180 (0.446)	0.686
**FEV**_**0.5**_	BPD	0.111 (0.342)	0.745
	GA	0.186 (0.119)	0.117
	***Vent days***	***−0.011 (0.003)***	***<0.001***
	***Z-BL***	***1.315 (0.365)***	***<0.001***
	PROM	−0.538 (0.511)	0.097
**FVC**	BPD	−0.083 (0.241)	0.731
	GA	0.126 (0.110)	0.252
	***Vent days***	***−0.005 (0.002)***	***0.009***
	***Z-BL***	***0.661 (0.300)***	***0.027***
	PROM	−0.139 (0.525)	0.792
**FEF**_**25–75**_	BPD	−0.087 (0.251)	0.730
	GA	0.063 (0.084)	0.454
	***Vent days***	***−0.007 (0.002)***	***0.001***
	***Z-BL***	***0.552 (0.214)***	***0.010***
	PROM	−0.487 (0.357)	0.173

BPD, severity of bronchopulmonary dysplasia; GA, gestational age; Vent days, total number of days on positive-pressure ventilation; Z-BL, z score of body length at the time of examination; PROM, history of premature rupture of membrane.

V’max_FRC_, maximal flow at functional residual capacity; FEV_0.5_, forced expiratory volume at 0.5 s; FVC, forced vital capacity; FEF_25–75_, forced midexpiratory flow.

## Discussion

The clinical condition of survivors of BPD generally improves over time, with symptoms becoming less severe; however, respiratory symptoms may persist into preschool years^16^. This study along with other studies of either previous “old” and present “new” BPD population, have revealed that such survivors experience persistent impairment in lung function^6,7,11^. Our study had shown some initial derangements in lung function (e.g., low respiratory system compliance and increased airway resistance) progressively recovered by 2 years of age. However, significant airflow limitation—measured as tidal and raised-volume forced expiration and low tidal volume—remained unchanged in children with severe BPD. Furthermore, our multivariate GEE model indicated that the number of days on positive-pressure ventilation was significantly inverse associated with z-scores of the majority of lung-function parameters in the later life. Performance of raised-volume flow-volume curve was also positively related to z-score of body length of preterm infants. Interestingly, current definition of BPD severity of preterm infants can’t well predict the outcome of their lung function in later life.

Progress in perinatal intensive care has enabled the identification of characteristic pathological findings of new BPD, which are a reduced number of alveoli with relatively simple and large structures and dysplasia of the pulmonary microvasculature^17^. Poor alveolarization originates from the incomplete deposition of the parenchymal elastin fiber^18^, which in turn leads to decreased parenchymal elasticity, increased tendency of airway closure, and increased peripheral airway resistance. This further results in lower compliance and higher resistance of the respiratory system in early age^7,19^. These insufficiencies gradually recover by 2 years of age (as observed in this study among children with mild to moderate BPD), indicating that alveolarization steadily occurs during growth. However, no improvement in respiratory mechanics was seen among children with severe BPD. Similar result was also noted among patients with moderate to severe BPD in Thunqvist’s report^19^. From the opinion of Barker’s hypothesis^20^, the deficit of lung function in young age would result in early onset of respiratory insufficiency in adulthood. Thus, long-term assessments are needed in these children to investigate the elasticity of peripheral lung parenchyma, such as those focusing on forced oscillation technique^21^.

Flow and volume measurements during forced expiration in children and adolescents born preterm have revealed the obstructed spirometric patterns in individuals with and without BPD^22–24^. To mimic spirometric measurement, assessments of forced flows and volumes in infancy are usually achieved through raised-volume rapid thoracoabdominal compression. In this study, airflow limitation was evident during early life, and the obstructed patterns were further exacerbated by 24 months of age; this phenomenon of airflow limitation was prominent and persistent in children with severe BPD. These findings indicate that the observed impairment in spirometric evolution is the manifestation of an early and persistent airway remodeling process occurred from infancy through childhood, and even adolescence.

V’max_FRC_ is measured during forced tidal expiration through rapid thoracoabdominal compression. Because this technique relies on functional residual capacity (FRC) as a landmark, the measurement results may vary widely among young infants^25^. Nevertheless, such variation can be controlled if the techniques are executed by experienced laboratory professionals following strict quality control measures^15,26^. Filippone and Owens *et al*. reported that the z-scores of V’max_FRC_ in infancy were highly related with those of spirometry in school-age children till young adults^27,28^. In the present study, although major differences were not observed at 6 months of age in infants with BPD, the z-scores decreased significantly in their later life (until 24 months). Thus, V’max_FRC_ is a reliable index for identifying older infants at risk of long-term lung function impairment.

Respiratory health and function in adulthood are closely related to lung function in childhood^29,30^. Although alveolarization continues throughout childhood, the outcomes are influenced by prenatal and early postnatal characteristics. Therefore, the lung function trajectories of adults appear to be partially determined very early in life. Both preterm birth and tobacco exposure have previously been associated with persistently low lung function performance during childhood and adolescence^31^. In a recent cohort study, preterm children with or without BPD exhibited a persistent decline in lung function after 4 years of age^32^. However, the determinants of lung function evolution among preterm infants had not previously been investigated. Our study clarified lung function evolution in such preterm infants. Impaired body length growth impedes lung function performance during growth. Sanchez-Solis *et al*. recently also shown that the gain of body length is associated with increase in lung function (exam of raised-volume forced expiration)^33^. This may related to the fact that body length is the dominant determinant of lung function in infants and preschool children^14,15,34^. Furthermore, our study found that postnatal respiratory morbidity (ventilator days) is significantly related to the performance of lung function (especially in exams of forced expiratory flow) in their later life up to preschool age. These results are consistent with the theory of continuous airway remodeling during infancy.

This study has some limitations that may have influenced the findings relating to comprehensive lung function trajectory in preterm infants. First, because this was a cohort study and not a case–control study, only a few patients had undergone all examinations at all time points. Therefore, obtaining well-defined trajectories of lung function evolution in individual preterm infants was difficult. Second, the airflow limitation may be affected by airway abnormalities, such as tracheobronchomalcia and congenital airway anomalies. Although chest computed tomography or bronchoscopy were not routinely performed in the study, infants with clinically evident airway anomalies were initially excluded from current study, thus minimizing the confounding effects. Third, several factors, such as preterm, severe postnatal respiratory infection, and maternal smoking, may be associated poor respiratory performance in later life. In this study, we had excluded infants with severe postnatal respiratory infection. In addition, although studies have demonstrated that maternal smoking unfavorably affects the respiratory function and health of infants^35,36^. This might have biased our results because prenatal tobacco exposure was not considered in our analysis. Nevertheless, only 3% of mothers of our study cohort reported smoking during pregnancy, and our previous study did not reveal any effect of maternal smoking on lung function^13,14^.

In conclusion, the poor lung-function performance of preterm infants can persist until late infancy, especially in infants with severe BPD. The (poor) gain in body length influences the (poor) lung function in infancy. Instead of current definition of BPD severity, the growth of body length at the time of exams (z-score of body length) and postnatal respiratory morbidity (the total number of days on positive-pressure ventilation) to be the main factors affecting the evolution of lung function, especially forced expiratory flow, in later infancy.

## Methods

### Study population and data collection

The present study was part of an ongoing larger, prospective, population-based birth cohort study called the Prediction of Allergies in Taiwanese Children (PATCH)^14,15,37,38^, which was initiated in 2013. Data collection is during the period of 2014 to 2018. Prematurely born (less than 36 weeks of gestation age) infants with or without BPD were recruited. Infants with major birth defects or congenital structural anomalies of the upper airway, those who were haemodynamically unstable, and those with a history of severe lower airway infection were excluded. Control participants, for the establishment of normal healthy reference, were simultaneously enrolled in the same period^14,15^. This study was approved by the Institutional Review Board of Chang Gung Memorial Hospital (IRB reference numbers 100-0286B and 103-6582A3). Our study was performed in line with Declaration of Helsinki and International Committee of Harmonisation good clinical practice. All the written informed consents were provided from the parents or legal guardians of neonates.

Detailed prenatal, perinatal, and postnatal data of the infants were collected from medical records. Measurements of lung function were performed at 6, 12, 18, and 24 months of corrected age. A diagnosis of BPD was made on the basis of the need for oxygen supplement at 28 days of age, and severity was determined at 36 weeks of gestation as follows:

Mild BPD: breathing room air.

Moderate BPD: requires < 30% FiO_2_ supplementary oxygen.

Severe BPD: requires ≥ 30% FiO_2_ supplementary oxygen and/or continuous positive airway pressure or ventilator use^39^.

### Infant lung function testing

Measurements were performed in healthy infants without respiratory tract infection for at least 3 weeks. Infant lung function testing was performed using the Jaeger Masterscreen BabyBody Paediatrics System (CareFusion, Hoechberg, German). The equipment conforms to the American Thoracic Society/European Respiratory Society (ATS/ERS) recommendations^40–44^. Group means of lung function index of preterm infants were compared with the data of normal healthy full-term infants (i.e., control infants) reported earlier^14,15^.

Detailed procedures and data collection methods involved in tidal breathing analysis, respiratory mechanics, forced tidal expiration, and raised-volume forced expiration have been reported previously^14,15^. The ratio of time to peak expiratory flow and total expiratory time (T_pef_/T_e_) in tidal breathing analysis, the resistance and compliance of the respiratory system (Rrs and Crs, respectively) in respiratory mechanics, and V’max_FRC_ in forced tidal expiration were determined for subsequent analysis. In the measurement of maximal raised-volume forced expiratory flow-volume curve, forced vital capacity (FVC), forced expiratory volume at 0.5 s (FEV_0.5_), and forced midexpiratory flow (FEF_25–75_) were determined for later analysis.

### Statistical analysis

Patient characteristics were recorded as means and standard deviations (or ranges) or numbers and proportions (%). Between-group comparisons were performed using Student’s t-test for continuous variables and the Fisher exact test for categorical variables.Fewer case number was found in mild (n = 18) and moderate BPD (n = 16) groups. Besides, similar grouping was frequently found in previous studies. So we combined two groups. We Since fewer case number was found in mild and moderate BPD group, we combined mild and moderate BPD group for later analysis. This grouping was also frequently found in previous reports. Recent studies have ever claimed that reference equations for infant respiratory function are laboratory- and ethnicity- specific^45,46^. Therefore, Currently, the Jaeger Masterscreen BabyBody is the only available commercial equipment and has been used worldwide. Lum *et al*. have recently revealed significant differences between the references of ‘in-house’ and commercial equipment the absolute values of individual test results from various infant lung function tests were recorded and converted into z-scores by using the our local reference equation^7,8^. Multivariable Generalized estimating equations (GEE) models were used to estimate the association of clinical determinants and lung-function parameter. A p value of <0.05 was considered statistically significant. All analyses were performed using IBM SPSS software version 20 (Armonk, NY, USA).
